# Application of Galvanostatic Non-Linear Impedance Spectroscopy to the Analysis of Metallic Material Degradation

**DOI:** 10.3390/ma17204985

**Published:** 2024-10-12

**Authors:** Pawel Slepski, Husnu Gerengi, Dominika Parasinska, Lukasz Gawel

**Affiliations:** 1 Department of Electrochemistry, Corrosion and Materials Engineering, Faculty of Chemistry, Gdansk University of Technology, 11/12 G. Narutowicza Str., 80-233 Gdansk, Poland; dominika.parasinska@pg.edu.pl (D.P.); lukasz.gawel@pg.edu.pl (L.G.); 2 Corrosion Research Laboratory, Department of Mechanical Engineering, Faculty of Engineering, Düzce University, 81620 Düzce, Turkey; husnugerengi@duzce.edu.tr

**Keywords:** degradation of metallic materials, non-linear electrochemical impedance spectroscopy, non-destructive corrosion monitoring

## Abstract

This study presents a novel application of Non-Linear Electrochemical Impedance Spectroscopy (NLEIS) in galvanostatic mode for the rapid, non-destructive assessment of metal degradation. By using galvanostatic mode instead of traditional potentiostatic methods, polarization-related challenges are mitigated, enabling more accurate and reliable analysis. The technique allows for the determination of corrosion rates (corrosion current) and material susceptibility to oxidation (Tafel coefficient) through a single measurement with a modulated AC perturbation signal. Theoretical assumptions of the method were validated through tests on both a non-linear model system and an experimental system. The proposed research methodology is highly effective for monitoring the condition of metallic materials in various environments, covering both anodic and cathodic processes.

## 1. Introduction

The degradation of metallic materials is a primary cause of various failures in structures and industrial equipment. The global cost associated with this phenomenon exceeds 3% of the global GDP [1]. Solutions involving the appropriate selection of materials and protective techniques are not always feasible due to technological or economic constraints.

In such cases, the most effective approach is the preventive monitoring of material degradation. The deterioration of metallic materials is predominantly related to electrochemical corrosion [2]. Consequently, electrochemical techniques based on current–voltage relationships are commonly used to analyze and monitor the progression of this process [3]:

(1) $$∆I=i_{corr}\left[exp\left(b_{a}∆E\right)-exp\left(-b_{c}∆E\right)\right]$$

where: ΔI—the response of the generated current [A], i_corr_—corrosion current [A], ∆E = E − E_corr_—polarization (difference of the actual (E) potential and the corrosion potential (E_corr_) [V], $b_{a}=\frac{ln(10)}{\beta_{a}}$, $b_{c}=\frac{ln(10)}{\beta_{c}}$, β_a_—Tafel slope coefficient of the anodic partial process, β_c_—Tafel slope coefficient of the cathodic partial process.

The simplest technique for determining the corrosion rate (i_corr_) and analyzing the anodic (β_a_) and cathodic (β_c_) processes is the Tafel analysis [4].

However, the deep polarization applied in this method (approximately ±250 mV) leads to irreversible changes in the material, preventing ongoing degradation monitoring. Using low polarization for linear current–voltage characteristic analysis (polarization resistance technique) avoids material damage but provides only indirect information [5,6].

These limitations are overcome by methods that use variable excitation signals, with sinusoidal perturbation being the most widely used.

Among the aforementioned methods we can distinguish:

- A method utilizing single sinusoidal excitation in the form of harmonic analysis (HA) proposed by Meszarosz et al. [7,8]. This technique has been applied in various corrosion studies [9,10,11].
- A method employing dual-frequency excitation, known as Electrochemical Frequency Modulation (EFM) [12,13,14].
- A method utilizing multiple frequency signals, generated either sequentially [15,16] or simultaneously [17,18,19].

The variable current method that provides the most comprehensive information about metal degradation is Non-Linear Electrochemical Impedance Spectroscopy (NLEIS) [20]. Its limitations related to long measurement time have recently been addressed through appropriately modified excitation signals [21,22].

Virtually all corrosion monitoring methods used to date operate in potentiostatic mode, assuming constant potential during electrochemical degradation. However, the decay of metallic materials is a non-stationary process where the corrosion potential undergoes significant fluctuations. A better alternative is the use of a method operating in galvanostatic mode.

In this paper, the authors introduce a novel approach to investigating corrosion processes by employing Non-Linear Electrochemical Impedance Spectroscopy in the current mode. This method is proposed as a promising approach for corrosion monitoring and a viable alternative to conventional techniques, offering a unique and valuable perspective on corrosion phenomena. By utilizing current perturbation signals, this approach facilitates a more nuanced and dynamic analysis of corrosion processes, which is crucial for advancing the field of materials science. The potential impact of this method on industrial and research applications is considerable, offering enhanced precision in corrosion monitoring and contributing to the development of more robust materials.

## 2. Method Description

The main problem, which occurs in corrosion analysis for measurements obtained in galvanostatic mode is the relation, which easily connects the voltage response signal with the current perturbation signal. Equation (1), which presents relation ∆I = f (∆E) could not be directly transformed into the form ∆E = f (∆I). The solution requires the introduction of one of three assumptions:

- $b_{c}=b_{a}=b$:

(2) $$∆E=\frac{1}{b}ln\left(\frac{∆I+\sqrt{{∆I}^{2}+4{i_{corr}}^{2}}}{2i_{corr}}\right)$$

- $b_{c}\gg b_{a}$:

(3) $$∆E={\frac{1}{b}}_{c}ln\left(i_{corr}\right)-{\frac{1}{b}}_{c}ln\left(i_{corr}-∆I\right)$$

- $b_{a}\gg b_{c}$:

(4) $$∆E={\frac{1}{b}}_{a}ln\left(∆I+i_{corr}\right)-{\frac{1}{b}}_{a}ln\left(i_{corr}\right)$$

The first solution pertains to situations in which the Tafel coefficients, both anodic and cathodic, are exactly the same. In practice, such cases are rather rare. The next solution concerns corrosion processes controlled by the anodic reaction. Such a situation, for example, occurs with materials that undergo passivation. The third case describes the corrosion process in which the rate of the phenomenon is determined by the cathodic reaction. This is the most commonly occurring form of the corrosion process and, for this reason, it has been chosen for further description.

### 2.1. Galvanostatic NLEIS for Cathodic Control Processes

If the current perturbation signal of Equation (4) will be changed to an AC signal in the form:

(5) $$∆I=∆I_{0}cos\left(\omega t\right)+I_{\mathrm{DC}}$$

where: *I_DC_* = 0—conditions of general corrosion.

The polyharmonic voltage response signal will take the form, described in Equation (6):

(6) $$∆E={∆E}_{0}+∆E_{1}cos\left(\omega t+\varphi \right)+∆E_{2}cos\left(2\omega t+2\varphi \right)+∆E_{3}cos\left(3\omega t+3\varphi \right)+∆E_{4}cos\left(4\omega t+4\varphi \right)+\cdots$$

where the amplitudes Δ*E_n_* of successive harmonics will be a function of the parameters *b_a_*, *i_corr_*, and Δ*I*_0_ (Equation (7a–c)):

(7a) $${∆E}_{1}=\frac{1}{b_{a}i_{corr}}{∆I}_{0}+\frac{1}{4b_{a}{i_{corr}}^{3}}{{∆I}_{0}}^{3}+\frac{1}{{8b}_{a}{i_{corr}}^{5}}{{∆I}_{0}}^{5}+\cdots$$

(7b) $${∆E}_{2}=-\frac{1}{{4b}_{a}{i_{corr}}^{2}}{{∆I}_{0}}^{2}-\frac{1}{8b_{a}{i_{corr}}^{4}}{{∆I}_{0}}^{4}-\frac{5}{{64b}_{a}{i_{corr}}^{6}}{{∆I}_{0}}^{6}-\cdots$$

(7c) $${∆E}_{3}=\frac{1}{{12b}_{a}{i_{corr}}^{3}}{{∆I}_{0}}^{3}+\frac{1}{16b_{a}{i_{corr}}^{5}}{{∆I}_{0}}^{5}+\cdots$$

The EIS technique is based only on the analysis of the fundamental harmonics of the response signal described by Equation (7a). Higher harmonics were not analyzed in the presented work. Based on this equation, the dependence of the Faradaic resistance *R_ct_* as a function of the amplitude of the perturbation signal Δ*I*_0_ can be described as Equation (8a,b):

(8a) $$R_{ct}=\frac{{∆E}_{1}}{{∆I}_{0}}$$

(8b) $$R_{ct}=\frac{1}{b_{a}i_{corr}}+\frac{1}{4b_{a}{i_{corr}}^{3}}{{∆I}_{0}}^{2}+\frac{1}{{8b}_{a}{i_{corr}}^{5}}{{∆I}_{0}}^{4}+\cdots$$

The presented Equation (8b) indicates that the Faradaic resistance of the corrosion system, controlled by the cathodic process, depends on the amplitude of the current perturbation signal used in galvanostatic mode. The higher the value of the perturbation amplitude used, the higher the value of the Faradaic resistance R_ct_ that will be obtained. This dependency can be used to determine the values of the corrosion parameters, such as the corrosion current and the anodic process Tafel slope coefficient. For this purpose, an analysis of the dependency between the Faradaic resistance and the amplitude of the perturbation signal $R_{ct}=f\left(∆I\right)$, based on at least the first two terms of the polynomial Equation (8b) should be carried out.

### 2.2. Galvanostatic NLEIS for Model System

**Figure 1:**
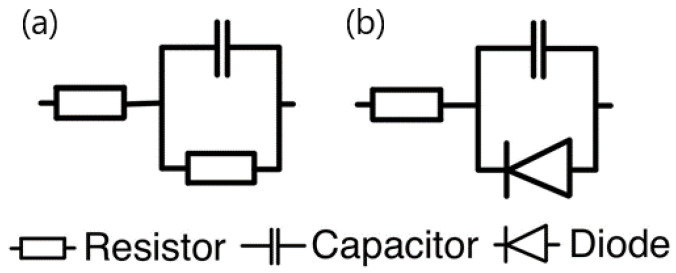
(**a**) Dummy cell with a resistor connected to a capacitor and resistor connected in parallel. (**b**) Dummy cell with a diode.

The verification of the correctness of research methods and measurement systems often relies on measurements using model systems. For impedance measurements of linear systems, dummy cells are commonly used, consisting of a resistor (in series) connected to a capacitor and resistor connected in parallel, as shown in Figure 1a [23].

In the case of simulating non-linear systems, the resistor is replaced with a diode, which is an element with a non-linear characteristic, as shown in Figure 1b [24]. The current–voltage relationship for a diode can be represented by a modification of the Shockley diode equation, which closely resembles the current–voltage behavior of an electrochemical system (Equations (9) and (10)):

(9) $$∆I=i_{s}exp\left(b_{d}∆E\right)$$

(10) $$∆E=\frac{1}{b_{d}}ln\left(\frac{∆I}{i_{s}}\right)$$

where: i_s_—saturation current, b_d_—Tafel slope coefficient for diode.

In this context, i_s_ is the equivalent of the corrosion current, while b_d_ corresponds to the inverse of the Tafel slope.

In the case of current excitation with a variable nature (Equation (5)), the amplitude of the voltage response for the fundamental harmonic can be described by a polynomial (Equation (11)):

(11) $$∆E_{1}=\frac{1}{b_{d}I_{DC}}{∆I}_{0}+\frac{1}{4b_{d}{I_{DC}}^{3}}{{∆I}_{0}}^{3}+\frac{1}{8b_{d}{I_{DC}}^{5}}{{∆I}_{0}}^{5}+\cdots$$

The above expression can be used to determine the diode resistance as a function of: excitation amplitude, current bias and voltage coefficient (Equation (12)):

(12) $$R_{d}=\frac{1}{b_{d}I_{DC}}+\frac{1}{4b_{d}{I_{DC}}^{3}}{{∆I}_{0}}^{2}+\frac{1}{8b_{d}{I_{DC}}^{5}}{{∆I}_{0}}^{4}+\cdots$$

It should be noted that the above equation is remarkably similar to Equation (8b) describing a corroding system under cathodic control conditions. This means that a substitute circuit based on the non-linear element, such as a diode, serves as an ideal means of validating the presented method.

## 3. Experimental

In the research, a dummy cell was used, consisting of a signal switching diode (part number: 1N414) connected in parallel with a 3.3 μF nominal value foil capacitor, with both components connected in series with a 10 Ω resistor. The current–voltage characteristic of the diode was determined through a linear voltage scan in the range of 0 to 0.7 V.

The measurements of the corrosion system under cathodic control were carried out in a 1M KCl solution (Sigma Aldrich, St. Louis, MO, USA) at a temperature of 32 °C stabilized by a LT ecocool 150 thermostat (Shepreth, Cambridgeshire, UK). Electrochemical measurements were made after conditioning the testing system for 24 h at the working temperature. The working electrode was composed of carbon steel (Fe 99.29% Mn 0.49% Ni 0.07% Cu 0.06% Cr 0.02%) in the form of a disk with a surface area of 1 cm^2^. The elemental analysis was performed by a Bruker (Brillerica, MA, USA) handheld XRF spectrometer (S1 Titan 600). The sample was immobilized in epoxy resin. Before measurements were taken, the surface was wet ground using a set of silicon carbide abrasive papers with a gradation from 400 up to 2500 grit, washed with deionized water, dried out and placed in an electrochemical cell. The reference electrode was a saturated calomel electrode (SCE) and the counter electrode was a platinum mesh.

The same card was used to record the total voltage and current signals from the investigated system. Dedicated software was developed using the LabView 2020 SP1 graphical programming environment by Emerson to control the measurement process, including signal generation, acquisition, signal recording, and subsequent analysis of the obtained impedance data.

**Figure 2:**
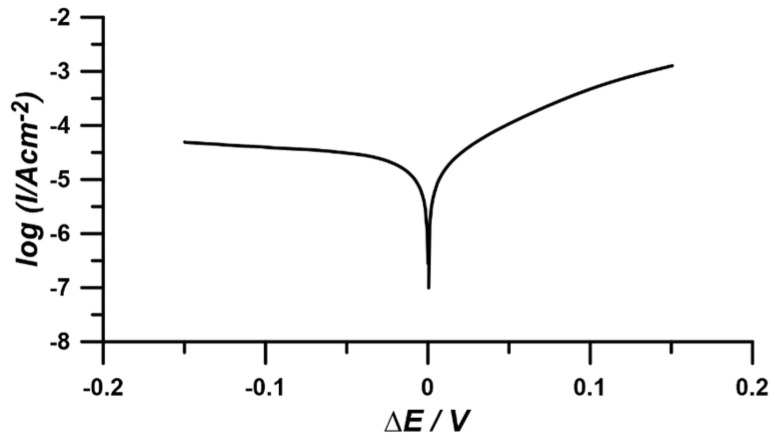
Current–voltage characteristics for determining corrosion current and Tafel coefficient via linear polarization at ±150 mV vs. *E_oc_* with a 1 mV/s scan rate.

The current–voltage characteristic, which served for the comparative determination of the corrosion current and Tafel coefficient *β_ɑ_*, was obtained through linear polarization of the working electrode in the range of ±150 mV vs. *E_oc_* at a scan rate of 1 mV/s (Figure 2).

**Figure 3:**
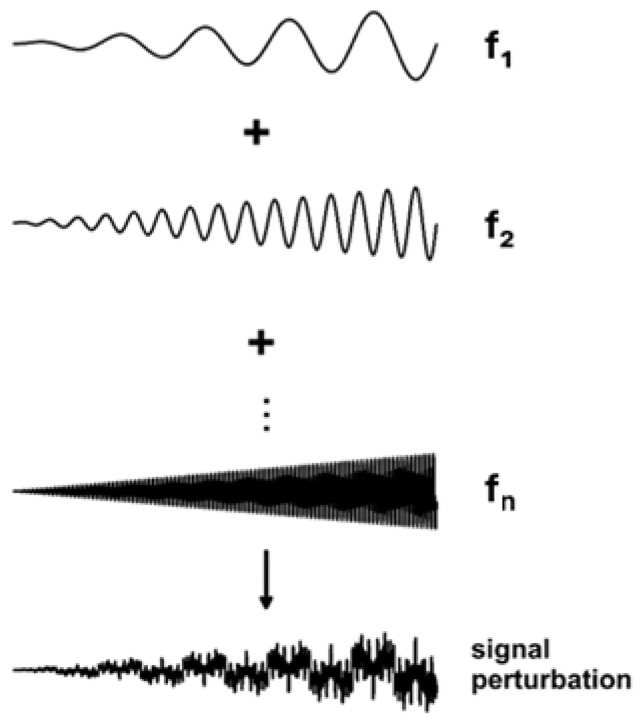
Scheme of generation of an AC signal perturbation.

Impedance measurements were conducted using a signal composed of elementary sinusoids with linearly varying amplitudes (Figure 3).

Impedance measurements for the non-linear model system were conducted at ten frequencies ranging from 1.5 kHz to 3 Hz. A constant current excitation amplitude of 32 µA was used, along with a linear continuously changing perturbation amplitude ranging from 0 µA to 15 µA at the rate of 1 µA/s. Variable current measurements for the corrosion system were performed at nine frequencies in the range of 100 Hz to 30 mHz, with a linear continuously changing perturbation amplitude ranging from 0 µA to 50 µA at the rate of 50 nA/s. The DC component, I_DC_, was set to 0 A (measured at the corrosion potential). Both the memory effects and hysteresis do not occur in this case.

## 4. Results and Discussion

### 4.1. Model System-Verification of the Method

**Figure 4:**
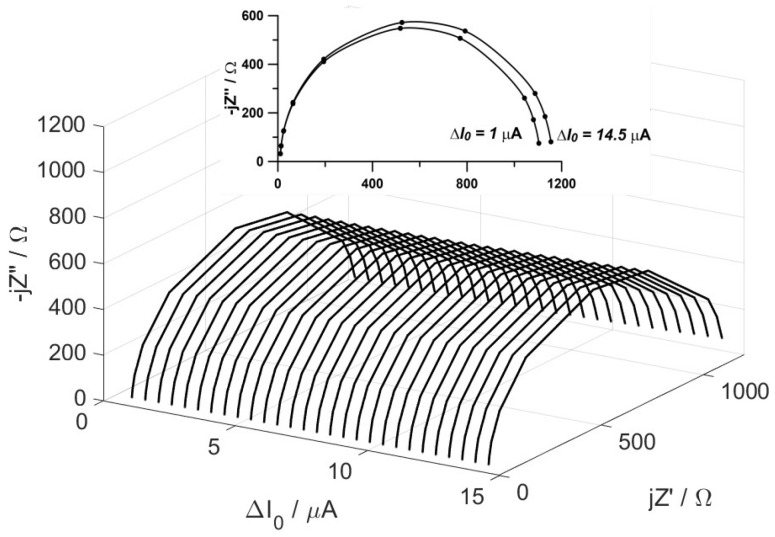
Changes in the impedance of the model system as a function of the amplitude of variable current excitation.

The impedance of the equivalent circuit obtained at a constant current I_DC_ = 32 μA and with variable components’ amplitudes, ${∆I}_{0}$ ranging from 0 to 15 μA is presented in Figure 4.

**Figure 5:**
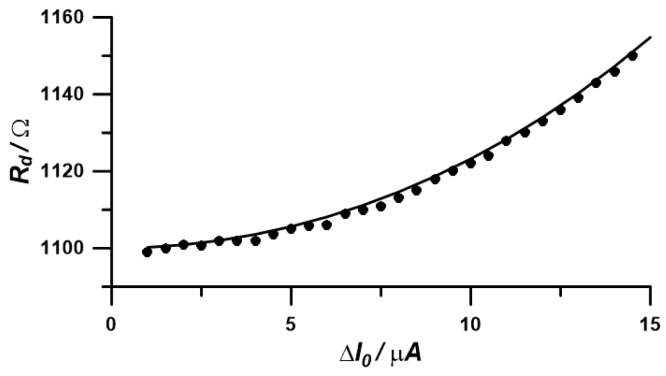
Changes in diode resistance as a function of the amplitude of variable current excitation.

The individual spectra exhibit semicircular shapes, which gradually enlarge as the amplitude of the variable excitation signal increases. The most significant changes in impedance are observed in the range of the lowest analyzed frequencies. By analyzing the individual impedance spectra using the Randles circuit R (CR), the variation in the diode resistance as a function of amplitude was obtained (Figure 5).

An increase in the variable current excitation from 0 µA to 15 µA results in an exponential increase in the obtained diode resistance, from approximately 1100 Ω to around 1160 Ω. This means that, according to Equation (12), by increasing the current amplitude of the variable excitation, we increase resistance values obtained based on impedance changes.

Based on the obtained current-resistance relationships, a non-linear regression was performed using the first two and the first three terms of Equation (12). The fitting results are presented in the table below.

**Figure 6:**
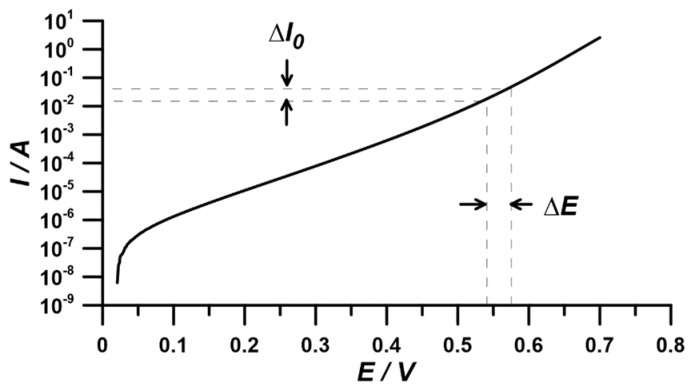
Current–Voltage characteristic of the diode used in the non-linear system model.

The experimental values (Table 1), both in the case of the polarization current and the diode voltage coefficient, are close to the real values. The magnitude of the error, which is approximately 5% in the case of fitting to the first two polynomial terms and around 10% in the case of fitting to three polynomial terms, largely depends on the range of amplitudes of the variable signal used for impedance measurement. In the analyzed case, the range of changes in the variable current single frequency amplitude was a maximum of 30 µA peak to peak (Figure 6).

The maximum voltage response generated as a result of the lowest single frequency is less than 37 mV peak to peak, a value which, in many impedance measurements, is considered as the linear range.

### 4.2. Cathodic Control-Real System

**Figure 7:**
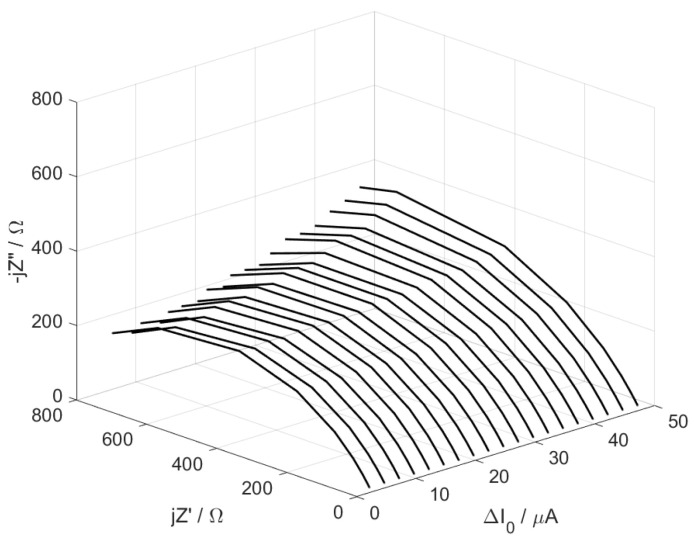
Impedance changes as a function of current perturbation amplitude ΔI_0_ for corrosion system under cathodic control.

The impedance changes for current perturbation amplitudes Δ*I*_0_ in the corrosion system under cathode control are presented in Figure 7. The current amplitude excitation was varied linearly from 0 μA to 50 μA. The individual spectra have the typical shape of a fragmentary semicircle. There is a marked increase in impedance as the amplitude of the variable excitation increases.

**Figure 8:**
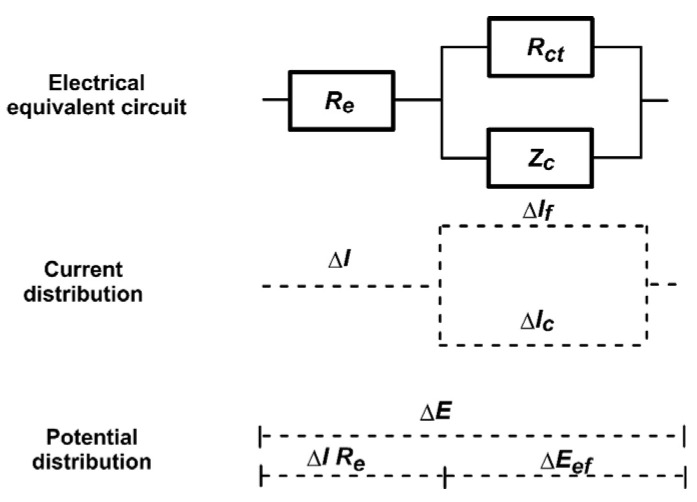
Electrical equivalent circuit, dependency of current distribution and dependency of the potential distribution of the tested system.

In order to use Equation (8a) to determine the corrosion current and anodic Tafel coefficient, it must be kept in mind that the *R_ct_* value is the ratio of the effective amplitude of the first harmonic appearing on the Faradaic element Δ*E_ef_* to the current perturbation signal flowing through the element Δ*I_f_*. In the tested system, an electrical double layer *Z_c_* and an electrolyte resistance *R_e_* can also be distinguished. An electrical equivalent circuit showing this dependency is shown in Figure 8.

In the first element, the resistance of the electrolyte (*R_e_*) of the electrical equivalent circuit influences the value of the voltage response signal on the Faradaic element R_ct_. In the second element, capacitance C_dl_, according to Kirchhoff’s law, influences the real value of the current perturbation signal on the Faradaic element R_ct_. Such a situation strongly implies harmonic analysis measurements when a single sinusoidal perturbation is used. In the case of using spectral measurements, the analysis of the obtained impedance spectrum gives the result in the form of the R_ct_ value, not under the influence of the Faraday current. Only in the case of significant influence of the electrolyte R_e_, when its value is close to the value of the R_ct_, should an appropriate correction of the values be included [21,24,25,26,27,28]

**Figure 9:**
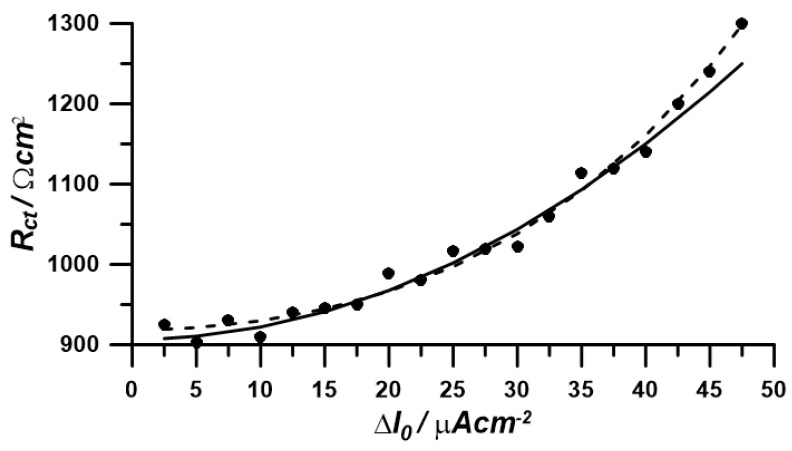
Characteristic of faradaic element *R_ct_* values changes in a function of amplitude changes of the current perturbation signal: (●)—experimental data (—)—fitting to the 2-term polynomial of Equation (11b) (- -)—fitting to the 3-term polynomial of Equation (11b).

The spectra shown in Figure 9 display slightly flattened semicircles instead of ideal ones. Therefore, to determine the Faradaic resistance (R_ct_), a simple three-element equivalent circuit R (QR) was employed. A constant phase element was used instead of capacitance due to both the energetic heterogeneity of the surface, as the material under investigation is an iron alloy containing additional elements, and the geometric irregularities resulting from the surface preparation, which does not produce a perfectly flat surface. Detailed information regarding surface distribution related to this phenomenon has been explained by Hirschorn et al. [29].

The obtained characteristic of R_ct_ changes in a function of perturbation amplitude changes R_ct_ = f (∆I_0_) is shown in Figure 7. In line with the earlier expectations, the course of the changes has the character of an increasing function.

To determine the corrosion parameters values from obtained data, such as corrosion current (*i_corr_*) and anodic Tafel coefficient (*β_a_*), it is required to perform a non-linear regression based on at least two terms of Equation (8b). Corrosion parameters were determined using the first two and the first three terms of the polynomial presented by Equation (8b). The results, along with the values obtained from classical polarization measurement are presented in Table 2.

**Figure 10:**
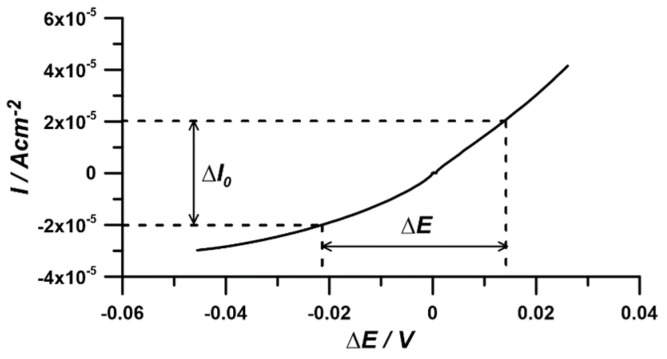
Amplitude-response signal relationship.

The values obtained by the NLEIS technique in the galvanostatic mode are similar to the values obtained in the classical Tafel extrapolation technique (see Table 2). The slight differences are probably the result of very low AC current perturbation Figure 10.

The current perturbation amplitude signals generate a small variable voltage response with an amplitude of less than 40 mV peak to peak. Such conditions mean that the results obtained by the polarization method relate to the measured sample significantly deviate from the equilibrium state.

## 5. Conclusions

Numerous electrochemical techniques exist for monitoring corrosion rates, but achieving reliable measurements while minimizing impact on the system remains challenging. Direct measurements of corrosion current often cause irreversible alterations or are skewed by electrical double layers and electrolyte resistance. Non-destructive methods usually provide only indirect, hard to correlate, results.

To address these limitations, the novel application of Non-Linear Electrochemical Impedance Spectroscopy (NLEIS) in galvanostatic mode offers a promising solution. This approach enables real-time, detailed insights into corrosion processes, making it a powerful tool for advancing the understanding and management of corrosion-related challenges. The use of NLEIS with current perturbation signals provides a unique and valuable perspective on corrosion phenomena. Moreover, the application of galvanostatic NLEIS with amplitude modulation shows great potential in scenarios where rapid changes in corrosion rates or non-stationarity of corrosion potential are critical factors. The adaptability and precision of this method make it an attractive option for researchers and practitioners seeking robust corrosion monitoring solutions. The proposed method represents a significant advancement in the field of corrosion science and technology, offering a novel approach that could lead to more accurate and reliable corrosion monitoring systems. By contributing to a deeper understanding of the relationships between material structure, properties, and functions, this technique aligns with the broader goals of materials research, facilitating the development of more durable and resistant materials across various applications.

## Figures and Tables

**Table 1:** Comparison of real values to experimentally obtained values.

Method	i_DC_ µA	*b_d_* 1/V
Real value	32.0	28.06
NLEIS-2 terms of the polynomial	33.7	26.98
NLEIS-3 terms of the polynomial	35.1	25.9

**Table 2:** Extrapolated corrosion rates and anodic Tafel slope for NLEIS and classic Tafel extrapolation for corrosion system under cathodic control.

Method	i_corr_ µA/cm ^2^	β_a_ mV
Tafel extrapolation	47.5	95
NLEIS-2 terms of the polynomial	38.6	81
NLEIS-3 terms of the polynomial	45.8	97

## Data Availability

The raw data supporting the conclusions of this article will be made available by the authors on request.
